# Multisite chronic pain: association with cognitive decline and post-mortem Alzheimer’s biomarkers

**DOI:** 10.1093/braincomms/fcaf208

**Published:** 2025-05-28

**Authors:** Tyler R Bell, Carol E Franz, Imanuel R Lerman, McKenna E Williams, Christine Fennema-Notestine, Matthew S Panizzon, Jeremy A Elman, David A Bennett, William S Kremen

**Affiliations:** Department of Psychiatry and Center for Behavior Genetics of Aging, University of California San Diego, San Diego, CA 92093, USA; Department of Psychiatry and Center for Behavior Genetics of Aging, University of California San Diego, San Diego, CA 92093, USA; Department of Anesthesiology, University of California San Diego, San Diego, CA 92093, USA; Department of Psychiatry and Center for Behavior Genetics of Aging, University of California San Diego, San Diego, CA 92093, USA; Department of Psychiatry and Center for Behavior Genetics of Aging, University of California San Diego, San Diego, CA 92093, USA; Department of Psychiatry and Center for Behavior Genetics of Aging, University of California San Diego, San Diego, CA 92093, USA; Department of Psychiatry and Center for Behavior Genetics of Aging, University of California San Diego, San Diego, CA 92093, USA; Rush Alzheimer’s Disease Center, Rush University Medical Center, Chicago, IL 60612, USA; Department of Psychiatry and Center for Behavior Genetics of Aging, University of California San Diego, San Diego, CA 92093, USA

**Keywords:** chronic pain, older adults, cognitive decline, *APOE* ɛ4, Alzheimer’s risk

## Abstract

Multisite chronic pain is a risk factor for Alzheimer’s disease, but its relationship with Alzheimer’s pathology remains unclear. This study examines the association between multisite chronic pain and single-site chronic pain with cognitive decline and post-mortem Alzheimer’s biomarkers in older adults. We conducted a longitudinal and prospective observational study using data from 3459 participants without dementia at baseline in the Religious Orders Study and Rush Memory and Aging Project. Participants (mean age = 77.94, SD = 7.93 years) were followed for an average of 8.53 years (SD = 5.74), with cognitive assessments and post-mortem biomarker analyses in a subset of brain donors (*n* range from 863 to 987 based on assay quality and tissue preservation). Cognitive function was measured using repeated assessments of five cognitive domains and a global composite score. Chronic pain was classified based on self-reported pain, determined by the number of joint sites affected during the first two assessments: Multisite chronic pain was defined as chronic pain in more than one site, single-site chronic pain as chronic pain in one site, and no chronic pain as no chronic pain sites. Brain beta-amyloid (Aβ) load and tau tangle density were assessed post-mortem, ∼10 months after the last clinical evaluation. This was assessed as a global measure across eight Alzheimer’s-affected brain regions, as well as specifically in the entorhinal cortex and hippocampus. *Apolipoprotein*-ɛ4 genotype was determined from blood and tissue samples. Compared to individuals with single-site (*n* = 506) or no chronic pain (*n* = 2599), participants with multisite chronic pain (*n* = 354) showed steeper declines in global cognition, episodic memory and working memory—especially among those with higher *apolipoprotein*-ɛ4 load—and were more likely to develop Alzheimer’s dementia. Multisite chronic pain was also linked to increased Aβ deposition in the entorhinal cortex and hippocampus, particularly in *apolipoprotein*-ɛ4 carriers. Compared to people with no chronic pain, single-site chronic pain did not differ on rate of cognitive decline, Alzheimer’s dementia risk or post-mortem Alzheimer’s biomarkers. Multisite, but not single-site, chronic pain is linked to steeper cognitive decline and increased Aβ deposition, particularly in individuals with elevated Alzheimer’s genetic risk. These findings suggest that reducing pain may mitigate the risk of cognitive decline and dementia.

## Teaser

Bell *et al*. report that multisite chronic pain in older adults is linked to faster cognitive decline and increased Alzheimer’s disease pathology, particularly among *APOE-ɛ4* carriers, whereas single-site pain is not associated with these outcomes. These findings support multisite pain as an independent risk factor for Alzheimer’s dementia.

## Graphical Abstract

**Graphical Abstract:**
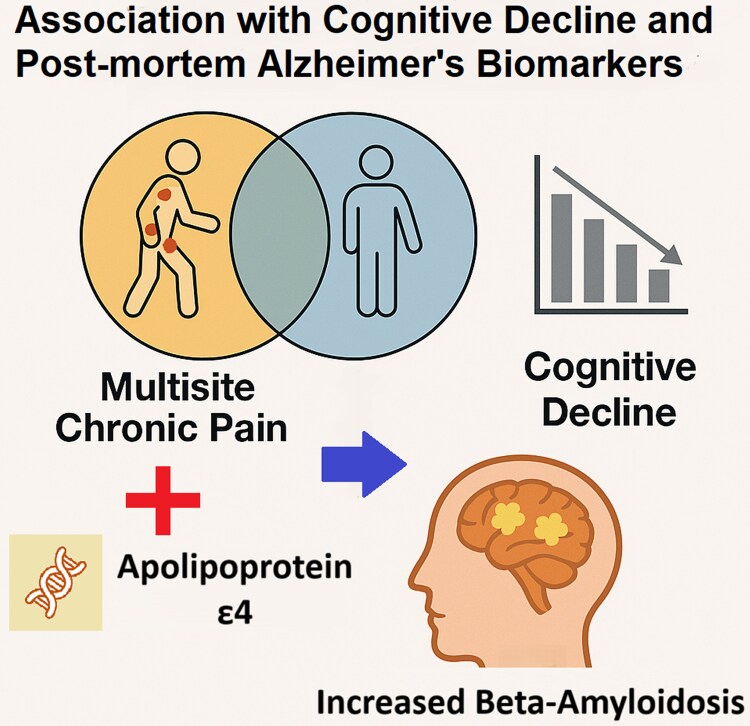

## Introduction

Chronic pain is defined as pain persisting or recurring beyond 3 months or more and affects about one in five older adults.^1,2^ The presence of chronic pain raises significant concerns for healthy aging, as research indicates it accelerates cognitive decline^3,4^ and doubles the risk of Alzheimer’s disease and related dementias.^5^ Additionally, chronic pain heightens the probability of other risk factors associated with Alzheimer’s disease, including emotional issues,^6-8^ functional decline^9^ and disruptions in sleep patterns.^10^ Beyond merely contributing to Alzheimer’s disease risk factors, there is evidence suggesting that chronic pain directly contributes to Alzheimer’s disease pathology. For instance, a mouse study demonstrated that inducing chronic pain through spared nerve injury increased tau protein expression in the hippocampus, along with hippocampal atrophy.^11^ A Mendelian randomization study in the UK Biobank established a causal link between chronic pain, memory decline and the risk of developing Alzheimer’s disease.^12^ Notably, this last study focused on MCP, suggesting the need to further investigate this chronic pain phenotype.

MCP refers to the experience of persistent or recurring pain in two or more anatomical locations.^13^ It is a prevalent geriatric syndrome, with studies indicating that around 10% of older adults may be affected at any one time.^14^ MCP is associated with mobility decline, reduced ability to conduct activities of daily living and a higher risk of falls and related disability.^13^ Like other chronic pain phenotypes, MCP has been linked to broader and steeper cognitive impairment, greater hippocampal atrophy and an increased risk of dementia.^15^ As such, more work is needed to examine how MCP is related to cognitive decline.

Considering the moderating role of the *APOE*-ɛ4 allele is crucial, as previous studies have shown that it amplifies the impact of risk factors on cognitive decline and the development of Alzheimer’s disease dementia.^16,17^ Although uncertain, the heightened risk may be attributed to the profound impact of the *APOE*-ɛ4 allele on brain structure and function directly and indirectly leading to Alzheimer’s disease pathology.^18^ Mechanisms include lipid-induced microglial dysfunction that fosters beta-amyloid deposition and hinders its clearance, promotes pro-inflammatory states and breaks down the blood–brain barrier.^19-21^ Consequently, individuals carrying the *APOE*-ɛ4 allele and experiencing MCP may be more vulnerable to cognitive decline and dementia than their non-ɛ4 carrier counterparts.

Our study aims to assess how MCP relates to cognitive decline and dementia risk in a large sample of older adults from the Religious Order Study/Memory and Aging Project (ROS/MAP). This builds and expands upon a recent study linking MCP to cognitive decline and hippocampal atrophy in the UK Biobank.^15^ In addition, we examine the modulatory role of the *APOE*-ɛ4 allele due to its established on Alzheimer’s disease pathology, which was not examined in the previous study. Our analyses focus on global cognitive ability as well as multiple cognitive domains affected by Alzheimer’s disease and aging. Overall, we hypothesize that older adults with MCP will show steeper cognitive decline, particularly in episodic memory and working memory–domains highly affected by Alzheimer’s disease pathology.^22^ We additionally explore changes in processing speed, semantic memory and perceptual orientation as well as overall risk of developing Alzheimer’s disease dementia. Our analyses also make use of post-mortem brain tissue to examine the association of MCP with primary indicators of Alzheimer’s disease pathology, including Aβ and tau tangles globally across Alzheimer’s disease-affected brain regions, and particularly in the hippocampus and entorhinal cortex—the most vulnerable sites of Alzheimer’s disease pathology.^23^

## Materials and methods

The ROS was established in 1994 and recruited nuns, priests and brothers from across the USA. The Rush MAP initiated in 1997 recruited community-dwelling persons in northeastern Illinois. Annual evaluations are conducted for both cohort studies by the same team and trainers with a large common core of identical data at the item level. ROS is conducted in 15 states across the USA and MAP in Cook and the Collar counties of Illinois. Institutional Review Board (IRB) approvals, informed consent, Anatomical Gift Act adherence and repository consent for data and biospecimen sharing are universal across both studies. Study procedures were approved by the IRB at the Rush University Medical Center, and all participants provided written informed consent. All consent and study procedures were conducted in alignment with the Declaration of Helsinki. Procedures for secondary data analysis were approved by the IRB at the University of California, San Diego.

A total of 3796 persons completed the baseline evaluation. Of these, we excluded 221 people with dementia at baseline and removed 116 individuals with missing pain data at baseline. This left 3459 eligible for our main sample, all of whom had cognitive data. Of this main sample, a sub-sample had data on *APOE*-ɛ4 status, post-mortem Aβ load and tau tangle density (*n*’s range from 863 to 987 based on assay quality and tissue preservation). The overall sample had a mean baseline age of 77.94 years (SD = 7.93) and a mean of 8.53 years (SD = 5.74) of follow-up.

Comprehensive documentation of data and associated materials, along with mechanisms for requesting data and biospecimens, is accessible through the Rush Alzheimer’s Disease Center Resource Sharing Hub (www.radc.rush.edu). The hub is continuously updated as data collection progresses, and it expands to accommodate new datasets available for sharing.

### Multisite and single-site chronic pain

Pain is a subjective experience best measured through self-report.^2^ In the ROS/MAP study, joint pain—the most common pain type among older adults^24^—was the sole pain source assessed. At baseline and during annual follow-ups, participants were asked, ‘Since your last interview on [last interview date], have you had pain or aching in any of your joints on most days for at least one month?’ Those who answered ‘yes’ were then asked to indicate the affected areas (neck, back, hands, hips or feet), with the option to select multiple locations.

Following established methods,^3,25^ we defined chronic pain using data from baseline through the first follow-up. This approach was chosen because our focus was on chronic pain as a risk factor for dementia—requiring categorization before the onset of dementia—rather than as a symptom. Moreover, assessing pain at later follow-ups could be problematic, as pain tends to be underreported once dementia develops.^26^ Using this method, we classified participants with MCP if they reported pain in the same location at both time points in more than one location. Participants reporting pain in only one location at both time points were classified as having single-site chronic pain (SCP). This categorization aligns with prior literature^13^ and the International Association for the Study of Pain’s (IASP) definition of chronic pain as persistent or recurrent pain lasting more than 3 months.^27^ Notably, our measure of MCP and SCP focused exclusively on the IASP-defined subtype of chronic musculoskeletal pain.^27^

### Neuropathological examination

Brain removal occurred, on average, 8.2 h post-mortem (SD = 5.1), ∼9.7 months after the last clinical evaluation. Quantification of Aβ-immunoreactive plaques and tau-immunoreactive tau tangles was conducted using computer-assisted sampling and immunohistochemistry. A monoclonal antibody 4G8 antibody (1:9000; Covance Labs, Madison, WI) directed at the *N*-terminus was employed to determine Aβ load (high affinity for Aβ42),^28^ while an anti-paired helical filament-tau antibody clone AT8 (1:2000; Thermo Scientific, Rockford, IL) was used to determine tau tangle density (high affinity for phosphorylated tau at Ser202/Thr205).^29^ Eight brain regions were assessed (mid-frontal cortex; inferior temporal; angular gyrus; calcarine cortex; anterior cingulate cortex; superior frontal cortex; hippocampus; and entorhinal cortex). As noted, our primary focus was on the hippocampus and entorhinal cortex as the primary sites for Alzheimer’s disease pathology.^23^ Results for a summary measure of all eight regions, global Aβ and tau tangle density, are also presented for comparison. Results for specific regions not of interest are provided in the Supplementary material.

### *APOE* genotyping

*APOE* genotype was determined by sequencing codons 112 and 158 of the *APOE* gene in DNA extracted from peripheral blood or frozen post-mortem brain tissue.^30^ For illustration purposes in Figures, *APOE*-ɛ4 carriers were defined as individuals with one or two copies of the ɛ4 allele; heterozygotes and homozygotes were grouped together. For analyses, an *APOE*-ɛ4 load was created which weights ɛ4 alleles on the genetic risk of Alzheimer’s disease, determined from a large genome-wide association study,^31^ while accounting for reduced risk due to the presence of ɛ2 alleles (equation: *APOE*-ɛ4 load = −0.47*number of e2 alleles + 1.12*number of ɛ4 alleles).

### Cognitive domains

Construct validation and test descriptions have been previously reported.^32^ Episodic memory was measured as a composite z-score of performances on word list repeat, word list recall, word list recognition, East Boston immediate recall, East Boston delayed recall, Logical Memory I (immediate recall) and Logical memory II (delayed recall) tests. Working memory was a z-scored composite of Digit Span Forward, Digit Span Backward and Digit Ordering scores. Semantic memory was measured using a z-score composite of scores on the Boston Naming Task, Category Fluency and Reading Test. Processing speed was measured as a z-score composite of Symbol Digit Modality Test, Number Comparison, Stroop Color Naming and Stroop Word Reading. Perceptual orientation was measured as a z-score composite of Line Orientation and Progressive Matrices (16 items). Composites were only calculated if not missing data on more than half of the measures. Global cognitive function was an averaged composite of episodic memory, working memory, processing speed, semantic memory and perceptual orientation.^32^

### Clinical diagnosis

Each participant’s cognitive status was determined through a three-step process. First, participants completed a battery of 19 neuropsychological test, which is used to estimate impairment across five cognitive domains. Next, a neuropsychologist—blinded to participant demographics—reviewed these ratings along with other clinical information to judge the presence of impairment or dementia. Finally, a clinician (neurologist, geriatrician or geriatric nurse practitioner) examined all available data and confirmed the diagnosis. Diagnoses of dementia and Alzheimer’s disease dementia followed National Institute of Neurological Disorders and Stroke–Alzheimer Disease and Related Disorders criteria,^33^ which require evidence of significant cognitive decline, including memory and at least one other cognitive area. People with other forms of dementia were also classified. Mild cognitive impairment (MCI) was diagnosed when some cognitive deficits are present but do not meet the criteria for dementia, while those without MCI or dementia are classified as being cognitively unimpaired. Clinical diagnosis was used into two ways for our investigation. First, we used diagnosis at their final wave of follow-up to determine if someone developed Alzheimer’s disease dementia prior to death. Second, we used clinical diagnosis to adjust for staging differences when looking at post-mortem Alzheimer’s disease biomarkers (described below).

### Covariates

Covariates for our analyses included sex, race, ethnicity, age at baseline and years of education reported at baseline, time-varying physical morbidities, time-varying depressive symptoms, time-varying analgesic use and total years completed. Participants’ sex was recorded as either male or female. Race was categorized into White, Black or African American, American Indian or Alaska Native, Native Hawaiian or Other Pacific Islander, Asian, Other or Unknown. Ethnicity was coded as Spanish/Hispanic/Latino, with options for Yes or No. Race/ethnicity status was coded for non-Hispanic White (coded 1), non-Hispanic Black (coded 2) and Hispanic (coded 3). The baseline age was calculated by subtracting the date of birth from the date of the baseline assessment and dividing it by the number of days per year (365.25). Years of education were recorded as the number of years of regular school reported at baseline. Physical morbidities were measured at baseline and follow-up waves. We specifically included history of stroke, congestive heart failure and diabetes as covariates as they are the leading morbidities related to poor cognition and dementia.^34,35^ Depressive symptoms are evaluated at baseline and follow-up using a modified 10-item version of the centre for epidemiologic studies depression scale.^36^ Participants indicate whether they experienced each of the 10 symptoms most of the time during the past week. The score represents the total number of symptoms reported. Items 4 and 7 are reverse coded so that a response consistent with a depressive symptom is scored as 1. At baseline and follow-ups, analgesic use was determined from a review of reported medications for presence of acetaminophen, aspirin, anti-inflammatories, glucocorticoids or opioids (Anatomical Therapeutic Chemical TC classes H02, M01A, N02A and N02B). To adjust for attrition bias, models also included number of total years completed as a covariate.

For autopsy analyses, we had additional covariates. This included age at death, which was calculated by subtracting the date of birth from the date of death and dividing the difference by the number of days per year (365.25) as well as the interval between death and autopsy (hours). We additionally included the interval from last assessment to death (years) and final clinical diagnosis (categorical variable with levels for cognitively unimpaired, MCI, Alzheimer’s disease dementia other dementia).

### Statistical analysis

First, we compared key covariates across individuals with no chronic pain, SCP and MCP. We then used linear mixed models with the GENLINMIXED package in SPSS version 28 (IBM Corp, Armonk, NY) to assess the association of chronic pain status (MCP, SCP and no chronic pain) with cognitive change. The models were set up as follows: Yij(Cognitive domain) ∼ β0ij + β1(chronic pain status)ij + β2(*APOE*-ɛ4 load)ij + β3(time [as time-varying age])ijk + β4(age at baseline)ij + β5(sex)ij + β6(race)ij + β7(education)ij + β8(number of years completed)ij + β9(analgesic use)ijk + β10(depressive symptoms)ijk + β11(stroke)ijk + β12(congestive heart failure)ijk + β13(diabetes)ijk + β14(chronic pain status*time)ijk + β15(chronic pain status *APOE-ɛ4 load)ijk + β16(*APOE*-ɛ4 load*time)ijk + β17(chronic pain status*time*APOE-ɛ4 load)ijk, nested within participant (i) and study (ROS or MAP, j) over time (k). Key terms of interest: β14(chronic pain status*time)ijk, assessing differential cognitive change rates and β17(MCP*time*APOE-ɛ4 load)ijk, assessing whether this association varies by *APOE*-ɛ4 load.

Next, we examined the association of chronic pain status with developing Alzheimer’s disease dementia before death using a logistic mixed model as follows: Probability of Yj(Alzheimer’s disease Dementia Status_final assessment_) ∼ β0ij + β1(chronic pain status)j + β2(*APOE*-ɛ4 load)j + β3(age_final assessment_)j + β4(age at baseline)j + β5(sex)j + β6(race)j + β7(education)j + β8(number of years completed)j + β16(analgesic use_final assessment_)j + β17(depressive symptoms_final assessment_)j + β18(stroke_final assessment_)j + β19(congestive heart failure_final assessment_)j + β20(diabetes_final assessment_)j + β21(chronic pain status*age at death)j + β22(chronic pain status*APOE-ɛ4 load)j, nested within study (ROS or MAP, j).

Lastly, we examined the association of chronic pain status with post-mortem pathology using linear mixed models set up as follows: Yj(Pathology) ∼ β0ij + β1(chronic pain status)j + β2(*APOE*-ɛ4 load)j + β3(age at death)j + β4(age at baseline)j + β5(sex)j + β6(race)j + β7(education)j + β8(number of years completed)j + β13(final cognitive diagnosis)j + β14(interval between death and autopsy)j + β15(interval between final assessment and death)j + β16(analgesic use_final assessment_)j + β17(depressive symptoms_final assessment_)j + β18(stroke_final assessment_)j + β19(congestive heart failure_final assessment_)j + β20(diabetes_final assessment_)j + β21(chronic pain status*age at death)j + β22(chronic pain status*APOE-ɛ4 load)j + β23(*APOE*-ɛ4 load*age at death)j + β24(chronic pain status*APOE-ɛ4 load*age at death)j, nested within study (ROS or MAP, j). Key terms of interest: β1(chronic pain status)j, assessing differential association with Alzheimer’s disease pathology; β21(chronic pain status*age at death), assessing moderation by age of death; and β22(chronic pain status*APOE-ɛ4 load)j, assessing moderation by *APOE*-ɛ4 load.

All analyses used full maximum likelihood to leverage available data, ensuring robust effect estimation and reducing bias from missing data. Statistical significance was set at α=0.05, with 95% confidence intervals calculated.

## Results

Descriptives are provided in Table 1. Overall, 14.6% (*n* = 506) reported SCP and 10.2% (*n* = 354) reported MCP from baseline to the first follow-up. People with SCP at the first follow-up continued to report SCP for 1.85 additional years (SD = 1.06); and people with MCP by the first follow-up wave continued to report MCP for 2.72 additional years (SD = 2.06). People with MCP were comparable to people without chronic pain or with single-site pain on baseline age, baseline cognitive status (unimpaired or MCI) and age at death (*P*’s > 0.05). People without chronic pain were more likely to have an *APOE*-ɛ4 allele (21.0%) compared to people with MCP (16.1%) or SCP (16.2%, *P* = 0.016). There were also differences such that the percent of female, the number taking analgesic medication and the level of depressive symptoms increased from no chronic pain, SCP to MCP (*P*’s < 0.05). People with MCP were more likely to be a race other than non-Hispanic White compared to no chronic pain and people with SCP (*P*’s < 0.05).

**Table 1:** Descriptive information of the overall sample by chronic pain status up to the first follow-up

						Differences across groups		MCP versus SCP	
		No chronic pain (*n* = 2599)	SCP (*n* = 506)	MCP (*n* = 354)	Overall (*n* = 3459)	*F*/χ²	*P*	*t*/χ²	*P*
Baseline age (years)	M (SD)	77.94 (7.93)	78.33 (7.64)	78.25 (7.49)	78.07 (7.83)	0.66	0.518	0.15	0.884
Age at death (years)	M (SD)	89.56 (6.67)	89.8 (6.73)	89.4 (6.50)	89.58 (6.65)	0.19	0.831	0.58	0.564
Missing	*n* (%)	1011 (38.9)	225 (44.5)	157 (44.4)	1393 (40.3)				
# of Years completed	M (SD)	8.53 (5.90)	8.64 (5.21)	8.56 (5.30)	8.53 (5.74)	0.03	0.971	0.26	0.796
Education (years)	M (SD)	16.44 (4.26)	16.38 (3.40)	16.06 (3.73)	16.37 (3.77)	1.32	0.269	1.27	0.206
Sex									
Female	*n* (%)	1823 (70.1)	408 (80.6)	314 (88.7)	2545 (73.6)	70.37	<0.001	10.06	0.002
Male	*n* (%)	776 (29.9)	98 (19.4)	40 (11.3)	914 (26.4)				
Race other than non-Hispanic White	*n* (%)	287 (11.0)	41 (8.1)	48 (13.6)	382 (10.8)	6.72	0.035	6.68	0.010
*APOE*-ɛ4 status									
Non-carrier	*n* (%)	1618 (62.3)	330 (65.2)	229 (64.7)	2177 (62.9)	8.30	0.016	0.01	0.993
Carrier	*n* (%)	547 (21.0)	82 (16.2)	57 (16.1)	686 (19.8)				
Missing	*n* (%)	434 (16.7)	94 (18.6)	68 (19.2)	596 (17.2)				
Initial diagnosis									
Unimpaired	*n* (%)	1906 (73.4)	387 (76.5)	268 (75.7)	2561 (74.0)	4.68	0.096	0.07	0.793
MCI	*n* (%)	695 (26.6)	119 (23.5)	86 (23.3)	898 (26.0)				
Depressive symptoms	M (SD)	0.73 (1.35)	1.14 (1.53)	1.79 (1.99)	1.05 (1.58)	46.01	<0.001	5.37	<0.001
Stroke	M (SD)	185 (7.1)	39 (7.7)	32 (9.0)	256 (7.4)	1.76	0.415	0.49	0.485
Congestive heart failure	*n* (%)	68 (2.6)	17 (3.4)	14 (4.0)	99 (2.9)	2.54	0.281	0.21	0.645
Diabetes	*n* (%)	326 (12.5)	76 (15.0)	55 (15.5)	457 (13.2)	4.12	0.127	0.04	0.836
Analgesic medication at baseline (yes)	*n* (%)	1802 (69.3)	405 (80.0)	314 (88.7)	2521 (72.9)	74.49	<0.001	11.40	<0.001

*APOE*-ɛ4, apolipoprotein epsilon 4 allele; MAP, memory aging project; MCI, mild cognitive impairment; MCP, multisite chronic pain; ROS, religious orders study; SCP, single-site chronic pain. Note age of death and *APOE*-ɛ4 status is only available on a sub-sample of participants.

### Cognitive decline

**Figure 1:**
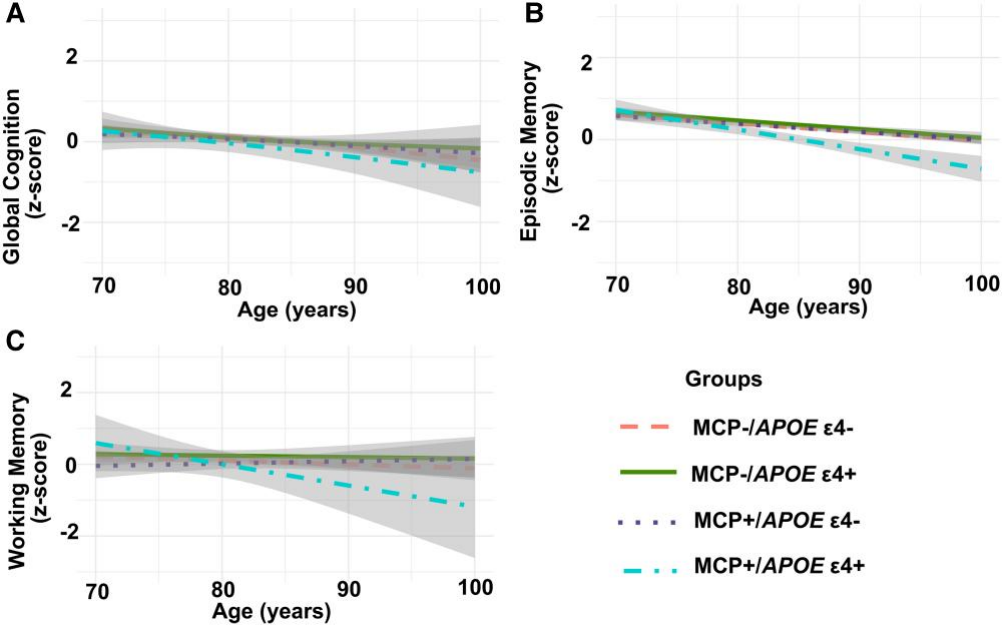
**The association of multisite chronic pain and *APOE*-ɛ4 allele status on global cognition (A), episodic memory (B) and working memory (C) as tested via linear mixed models**. Each data point on the line represents the group mean on the cognitive outcome with a 95% confidence interval (grey region). The original association was tested using linear mixed-effect models with a grouping variable indicating single-site, multisite and no chronic pain and an interaction with a variable indicating *APOE*-ɛ4 load. For illustration purposes, cognitive trajectories are plotted by MCP+/ɛ4− (*n* = 1949), MCP−/ɛ4+ (*n* = 629), MCP+/ɛ4− (*n* = 229) and MCP+/ɛ4+ (*n* = 57). Note. ɛ4+ = presence of ɛ4 allele; ɛ4− = absence of ɛ4 allele; MCP+= presence of multisite chronic pain. Although not everyone had ɛ4 status, model analyses are based on all available data (*n* = 3459). The MCP− group included people with no chronic pain or with single-site chronic pain. Linear mixed models showed that MCP+/ɛ4 + group had significantly steeper decline in global cognition, episodic memory and working memory compared to people with no chronic pain (*b*’s range from −0.01 to −0.02, *P*’s < 0.05) or with SCP (*b*’s range from = −0.03 to −0.01, *P*’s < 0.001).

Table 2 shows that individuals with MCP who carried at least one APOE-ɛ4 allele experienced a more rapid decline in global cognitive function compared to those with no chronic pain or with SCP. Specifically, when looking at the interaction between time and APOE-ɛ4 load, individuals with MCP showed a significantly steeper decline in global cognition compared to those with no chronic pain (*b*=−0.10, SE = 0.03, *P* = 0.001). In a similar comparison, MCP individuals also experienced a steeper decline than those with SCP (*b*=−0.01, SE = 0.004, *P* = 0.001). In contrast, individuals with SCP did not differ from those with no chronic pain in their rate of cognitive change, regardless of APOE-ɛ4 load (*P*’s > 0.05). A similar pattern emerged for episodic memory, working memory, processing speed and perceptual orientation as detailed in Table 2 and Supplementary Table 1. Associations with global cognition, episodic memory and working memory are illustrated in Fig. 1.

**Table 2:** Mixed model examining associations of chronic pain, time and APOE ɛ4 load on cognition (*n* = 3459)

	Global cognition			Episodic memory			Working memory		
Model term	b	SE	*P*	b	SE	*P*	b	SE	*P*
Time (time-varying age)	**−0.04**	0.**001**	**<0**.**001**	**−0**.**007**	0.**001**	**<0**.**001**	**−0**.**03**	0.**001**	**<0**.**001**
Main effects (baseline differences)									
MCP (versus no CP)	0.04	0.037	0.314	0.03	0.040	0.532	−0.02	0.042	0.722
SCP (versus no CP)	0.03	0.032	0.326	0.**08**	0.**035**	0.**018**	−0.02	0.036	0.620
MCP (versus SCP)	0.01	0.045	0.890	−0.06	0.049	0.232	0.003	0.051	0.954
*APOE*-ɛ4 load	**−0**.**13**	0.**021**	**<0**.**001**	**−0**.**08**	0.**025**	**<0**.**001**	−0.05	0.024	0.**055**
Interactions with *APOE*-ɛ4 load									
MCP (versus no CP)	0.**14**	0.**064**	0.**035**	0.07	0.078	0.356	0.12	0.074	0.100
SCP (versus no CP)	0.03	0.053	0.601	0.08	0.066	0.222	0.01	0.060	0.813
MCP (versus SCP)	0.11	0.078	0.166	−0.009	0.095	−0.926	0.11	0.089	0.229
Interactions with time									
MCP (versus no CP)	0.00	0.002	0.243	0.002	0.002	0.331	−0.002	0.002	0.343
SCP (versus no CP)	0.00	0.002	0.069	0.01	0.002	0.933	−0.001	0.002	0.480
MCP (versus SCP)	0.00	0.002	0.719	0.002	0.003	0.398	−0.001	0.003	0.794
*APOE*-ɛ4 load*time	**−0**.**02**	0.**001**	**<0**.**001**	**−0**.**01**	0.**001**	**<0**.**001**	**−0**.**01**	0.**001**	**<0**.**001**
Interactions with *APOE*-ɛ4 load and Time									
MCP (versus no CP)	**−0**.**01**	0.**003**	0.**004**	**−0**.**02**	0.**005**	**<0**.**001**	**−0**.**01**	0.**004**	0.**024**
SCP (versus no CP)	0.00	0.003	0.129	0.003	0.004	0.450	0.01	0.003	0.114
MCP (versus SCP)	**−0**.**01**	0.**004**	**<0**.**001**	**−0**.**03**	0.**006**	**<0**.**001**	**−0**.**02**	0.**005**	0.**003**

*APOE*-ɛ4, apolipoprotein epsilon 4 allele; CP, chronic pain; MCP, multisite chronic pain; SCP, single-site chronic pain. Models were adjusted for the effect of age at baseline, sex, education, race, analgesic use, number of total follow-ups completed, depressive symptoms, history of stroke, history of congestive heart failure and history of diabetes. Bolded values indicate statistically significant associations.

Across all models, older age at baseline (*b*’s range from −0.43 to −0.17), male sex (*b*’s range from −0.25 to −0.017), fewer years of education (*b*’s range from 0.11 to 0.18), being of a race/ethnicity other than non-Hispanic white (*b*’s range from −0.60 to −0.23), completed a fewer number of years (*b*’s range from 0.03 to 0.06) and time (*b*’s range from −0.06 to −0.01) were each associated with worse cognitive function (*P*’s < 0.05).

### Risk of Alzheimer’s disease dementia

Logistic linear mixed models assessed the likelihood of developing Alzheimer’s disease dementia by the final assessment. As shown in Supplementary Table 2, people with MCP had a higher likelihood of developing Alzheimer’s disease dementia compared to people without chronic pain (OR = 1.65, SE = 0.179, *P* = 0.005). People with MCP showed a higher likelihood of developing Alzheimer’s disease dementia compared to people with SCP although non-significant (OR = 1.43, SE = 0.213, *P* = 0.095). People with SCP did not show significant differences in the likelihood of developing Alzheimer’s disease dementia compared to people with no chronic pain (*P* = 0.312). There were no interactions of chronic pain status with APOE-ɛ4 load (*P*’s > 0.05). Regarding covariates, history of congestive heart failure was associated with higher likelihood of developing Alzheimer’s disease dementia (OR = 3.22, SE = 0.404, *P* = 0.004).

### Alzheimer’s disease pathology

**Figure 2:**
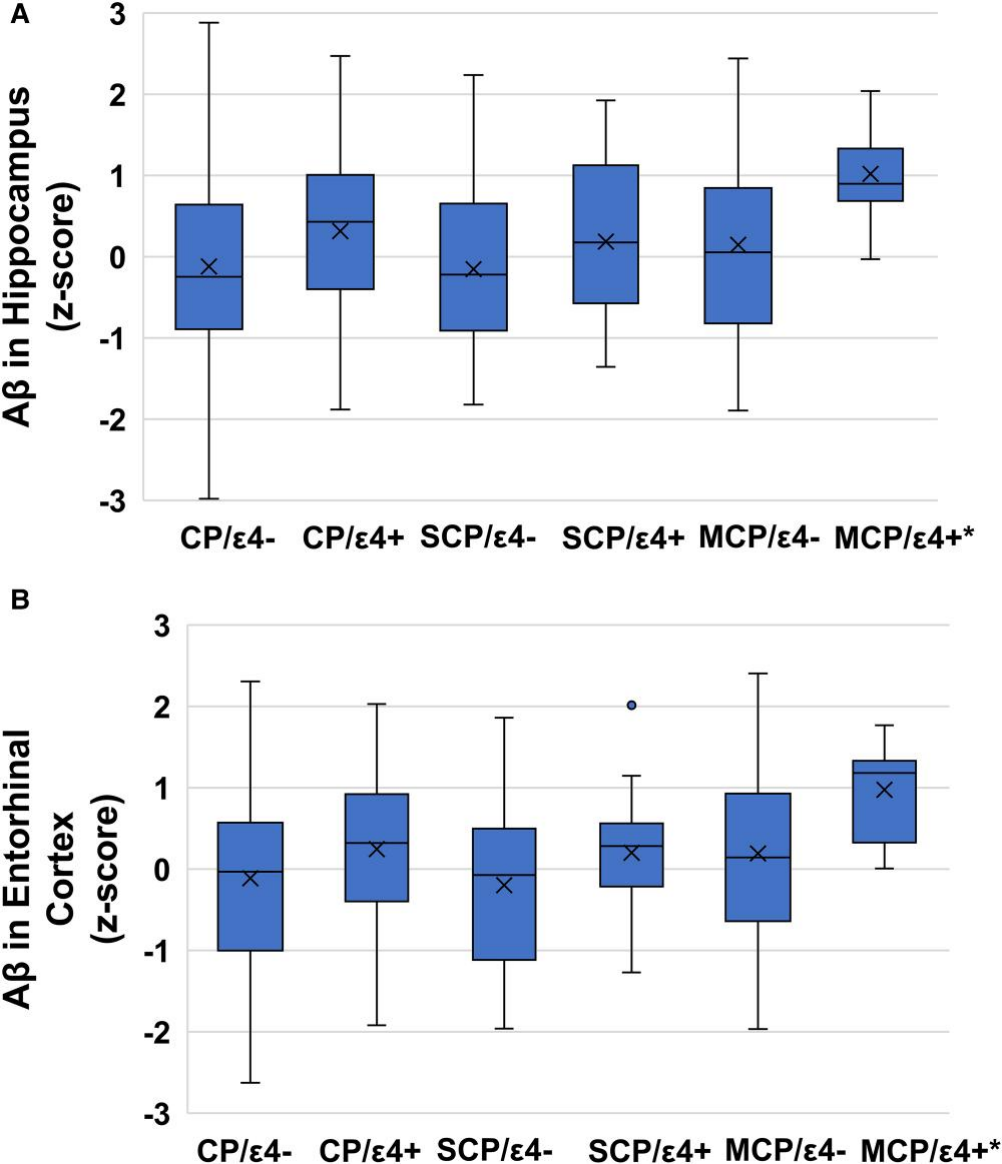
**Levels of β-amyloid (Aβ) in the entorhinal cortex (A) and the hippocampus (B) based on chronic pain and *APOE*-ɛ4 allele status**. Each box on the graph represents the middle 50% distribution of β-amyloid in each group with the mean denoted as X and median denoted as the line within the box. The whiskers represent the range of values in the group. The original association was tested using linear mixed-effect models with a grouping variable indicating single-site, multisite and no chronic pain and an interaction with a variable indicating *APOE*-ɛ4 load. For illustration purposes, differences in β-amyloid levels are plotted by CP-/ɛ4− (entorhinal: *n* = 402; hippocampus: *n* = 414), CP−/ɛ4+ (entorhinal: *n* = 149; hippocampus: *n* = 154), SCP+/ɛ4− (entorhinal: *n* = 142; hippocampus: *n* = 147), SCP+/ɛ4+ (entorhinal: *n* = 34; hippocampus: *n* = 36). MCP+/ɛ4− (entorhinal: *n* = 104; hippocampus: *n* = 107) and MCP+/ɛ4+ (entorhinal: *n* = 26; hippocampus: *n* = 26). CP, chronic pain; ɛ4+, presence of ɛ4 allele; ɛ4−, absence of ɛ4 allele; MCP, multisite chronic pain; SCP, single-site chronic pain. *There was a significant interaction such that this difference is magnified with older age at death. Linear mixed models showed that the MCP+/ɛ4 + group had higher levels of Aβ in the entorhinal cortex and hippocampus at older ages compared to people with no chronic pain (*b*’s range from 0.41 to 0.55, *P*’s < 0.05) or with SCP (*b*’s range from 1.17 to 1.77, *P*’s < 0.05).

Table 3 indicates that there were no overall differences in global Aβ or tau tangle density by chronic pain status, regardless of age or *APOE*-ɛ4 load (*P*’s > 0.05). However, regional analyses revealed that among older individuals with higher *APOE*-ɛ4 loads, those with MCP exhibited elevated Aβ levels in the entorhinal cortex and hippocampus compared to individuals with SCP or no chronic pain. Specifically, the interaction between MCP (versus no chronic pain) with APOE-ɛ4 load and age at death produced beta coefficients ranging from 0.41 to 1.55 (*P*’s < 0.05), and the comparison between MCP and SCP showed beta coefficients ranging from 1.17 to 1.77 (*P*’s < 0.05). These findings are illustrated in Fig. 2. Exploratory analyses (Supplementary Table 3) demonstrated a similar pattern for Aβ in the inferior temporal and calcarine cortices.

**Table 3:** Associations of chronic pain with aβ and tau tangles globally and in the entorhinal cortex and hippocampus

	Global Aβ (*n* = 922)			Aβ in EC (*n* = 857)			Aβ in Hip. (*n* = 884)		
Main effects	*b*	SE	*P*	*b*	SE	*P*	*b*	SE	*P*
MCP (versus no CP)	0.12	0.098	0.213	0.68	0.371	0.067	0.24	0.182	0.194
SCP (versus no CP)	−0.13	0.086	0.130	−0.12	0.327	0.722	−0.09	0.159	0.580
MCP (versus SCP)	**0.25**	**0.120**	**0.035**	0.80	0.452	0.078	0.32	0.221	0.143
APOE-ɛ4 load	**0.51**	**0.057**	**<0**.**001**	**1**.**18**	**0.215**	**<0**.**001**	0.63	0.105	<0.001
Interaction with age^a^									
APOE-ɛ4 load	−0.08	0.052	0.131	−0.25	0.206	0.225	0.03	0.098	0.777
MCP (versus no CP)	0.13	0.097	0.198	**0.75**	**0.372**	**0.043**	**0.42**	**0.180**	**0.021**
SCP (versus no CP)	−0.08	0.084	0.325	−0.26	0.323	0.414	−0.04	0.156	0.783
MCP (versus SCP)	0.21	0.119	0.082	**1**.**02**	**0.458**	**0.026**	**0.46**	**0.222**	**0.038**
Interaction with APOE-ɛ4 load									
MCP (versus no CP)	0.34	0.179	0.058	1.25	0.663	0.060	0.36	0.328	0.271
SCP (versus no CP)	0.05	0.137	0.708	0.55	0.522	0.293	0.34	0.251	0.179
MCP (versus SCP)	0.29	0.210	0.172	0.70	0.788	0.373	0.02	0.385	0.950
Interaction with APOE-ɛ4 load and age									
MCP (versus no CP)	0.35	0.190	0.066	**1**.**55**	**0.716**	**0.031**	**0.41**	**0.196**	**0.037**
SCP (versus no CP)	0.00	0.150	0.991	−0.22	0.566	0.693	−0.52	0.274	0.058
MCP (versus SCP)	0.35	0.232	0.129	**1**.**77**	**0.868**	**0.041**	**1**.**17**	**0.428**	**0.006**
	Global Tangles (*n* = 1016)			Tangles in EC (*n* = 961)			Tangles in Hip. (*n* = 987)		
Main effects	*b*	SE	*P*	*b*	SE	*P*	*b*	SE	*P*
MCP (versus no CP)	0.04	0.101	0.697	1.80	1.243	0.148	1.00	1.722	0.563
SCP (versus no CP)	−0.08	0.089	0.376	−0.01	1.087	0.996	−0.82	1.510	0.589
MCP (versus SCP)	0.12	0.123	0.340	1.80	1.513	0.233	1.81	2.101	0.389
APOE-ɛ4 load	**0**.**42**	**0.058**	**<0**.**001**	**3**.**66**	**0.708**	**<0**.**001**	**3**.**95**	**0.981**	**<0**.**001**
Interaction with age at death									
APOE-ɛ4 load	0.03	0.053	0.629	0.71	0.676	0.292	−0.35	0.910	0.702
MCP (versus no CP)	0.04	0.098	0.695	1.00	1.205	0.408	0.40	1.677	0.814
SCP (versus no CP)	0.09	0.085	0.312	0.52	1.058	0.620	2.63	1.453	0.070
MCP (versus SCP)	−0.05	0.120	0.695	0.47	1.483	0.750	−2.24	2.059	0.277
Interaction with APOE-ɛ4 load									
MCP (versus no CP)	−0.02	0.177	0.902	4.79	3.021	0.060	−4.68	2.989	0.118
SCP (versus no CP)	0.05	0.142	0.716	−1.35	1.749	0.441	−0.34	2.385	0.885
MCP (versus SCP)	−0.07	0.212	0.729	−3.44	2.584	0.183	−4.34	3.566	0.224
Interaction with APOE-ɛ4 load and age									
MCP (versus no CP)	0.12	0.199	0.546	−2.28	2.436	0.348	−1.94	3.404	0.570
SCP (versus no CP)	**−0**.**34**	**0.152**	**0.024**	**−4**.**15**	**1**.**852**	**0.025**	−4.46	2.551	0.081
MCP (versus SCP)	0.46	0.239	0.054	1.87	2.914	0.522	2.53	4.065	0.534

*APOE*-ɛ4, apolipoprotein epsilon 4 allele; CP, chronic pain; MCP, multisite chronic pain; SCP, single-site chronic pain. Models were adjusted for the effect of age at baseline, sex, education, race, analgesic use, number of total follow-ups completed, depressive symptoms, stroke, congestive heart failure and diabetes. ^a^Age refers to age at death. Bolded values indicate statistically significant associations.

Furthermore, as shown in Supplementary Table 4, people with MCP had higher Aβ levels in the anterior cingulate cortex and superior frontal cortex compared to people without CP (*b*’s range from 1.02 to 1.05, *P*’s < 0.05) and people with SCP (*b*’s range from 0.54 to 1.70, *P*’s < 0.01). These differences were not moderated by *APOE*-ɛ4 load (*P*’s > 0.05) but the Aβ differences between MCP and SCP in the superior frontal cortex was magnified at older ages (*b* = 1.33, *P* = 0.013). In contrast, as shown in Table 3 and Supplementary Tables 3 and 4 (as well as Supplementary Fig. 1), no regional differences in tau tangle density were observed by chronic pain status, regardless of age at death or *APOE*-ɛ4 load (*P*’s > 0.05).

Regarding covariates, fewer years of education (*b*’s range from −0.64 to −0.11), MCI (*b*’s range from 0.23 to 0.93) and Alzheimer’s disease dementia (*b*’s range from 0.54 to 2.37) were associated with higher Aβ load across models (*P*’s < 0.05). Additionally, greater years between last assessment and death (*b*’s range from 0.13 to 2.15) as well as Alzheimer’s disease dementia status (*b*’s range from 0.83 to 13.95) were linked to higher tau tangle density in the hippocampus and entorhinal cortex (*P*’s < 0.05).

## Discussion

MCP has been linked to elevated risk of cognitive decline, hippocampal atrophy and dementia.^15^ The present study advances our knowledge by assessing whether older adults with MCP experience differential rates of cognitive decline, risk of Alzheimer’s disease dementia and levels of Alzheimer’s disease pathology. We additionally explored whether the *APOE*-ɛ4 allele modulated these effects. We found that older adults with MCP who carried the *APOE*-ɛ4 allele exhibited steeper decline in global cognition than older adults without chronic pain. This was also found in specific domains of episodic memory, working memory, processing speed and perceptual orientation. Post-mortem analyses found that older adults with MCP who carried the *APOE*-ɛ4 allele had greater Aβ load particularly in the hippocampus, entorhinal cortex and inferior temporal lobe than those without chronic pain or with SCP.

Our findings of steeper cognitive decline among individuals with MCP add to previous studies. Three epidemiological studies have linked chronic pain to cognitive decline, limited by reliance on a cognitive screener. Whitlock *et al*.^3^ analysed data from 10 065 older adults in the Health and Retirement Study, showing that persistent pain was linked to a 9.2% steeper memory decline and a 7.7% increased likelihood of dementia over 10 years. Similarly, Rong *et al*.^37^ used data from 6869 participants in the English Longitudinal Study of Ageing and reported that persistent moderate to severe pain correlated with steeper declines in global cognitive scores over a median follow-up of 12 years. Furthermore, Zhao *et al*.^15^ found steeper cognitive decline in people with MCP compared to SCP and no chronic pain. Other studies, while lower in sample size, have linked chronic pain to cognitive decline using more detailed neuropsychological testing. Rouch *et al*.^38^ investigated 693 participants aged 65 and older from the PAQUID cohort, finding that chronic pain was significantly associated with poorer cognitive performance, particularly in processing speed, over a 15-year period. Bell *et al*.,^39^ using data from the ACTIVE trial, studied 688 participants with a mean age of 74 and found that chronic pain was associated with steeper declines in processing speed, memory and other cognitive domains over a decade. Our study adds to this literature by focusing on a unique phenotype, MCP, which has been associated with Alzheimer’s disease risk—and assessing cognitive decline up to 29 years. Furthermore, we found that associations were stronger among people with multiple chronic pain sites and who also carried the *APOE*-ɛ4 risk allele.

Our study found that MCP was associated with an increased risk of Alzheimer’s disease dementia. This finding is consistent with previous research linking both the number of pain sites and widespread pain to heightened dementia risk. For instance, Wang and Liu^40^ analysed data from the Framingham Heart Study, where 2464 participants were followed for a median of 10 years, and reported that widespread pain was linked to a 43% increased risk of all-cause dementia and a 47% increased risk of Alzheimer’s disease dementia. Similarly, Tian *et al*.^41^ examined 356 383 participants from the UK Biobank over a median follow-up of 13 years and found that each additional chronic pain site corresponded to an increased risk of all-cause dementia (HR = 1.08 per site) and Alzheimer’s disease dementia (HR = 1.09 per site). In addition, Zhao *et al*.^15^ assessed MCP and dementia risk directly in 354 943 UK Biobank participants and determined that MCP was associated with a 36% greater risk of dementia compared to pain-free controls. Our findings extend this literature by linking MCP to Alzheimer’s disease dementia risk specifically and by exploring its interaction with *APOE*-ɛ4 load. Although the interaction between MCP and *APOE*-ɛ4 load was not statistically significant, it showed a positive trend (OR = 1.12).

In addition, we looked at Alzheimer’s disease biomarkers, which have only been done in a few studies. Compared to individuals with no chronic pain or SCP, those with MCP exhibited higher Aβ in regions most vulnerable to Alzheimer’s disease pathology—including the entorhinal cortex, hippocampus and inferior temporal cortex—with levels further amplified by APOE-ɛ4 load and age. Additionally, MCP was associated with elevated Aβ in the anterior cingulate cortex and superior frontal cortex, independent of *APOE*-ɛ4 status and age. These findings suggest that MCP reflects an aetiology that may predispose individuals to increased Aβ accumulation in regions already susceptible to Alzheimer’s disease pathology. While early affected regions show an amplified Aβ burden in MCP as a function of Alzheimer’s disease genetic risk and aging, the elevated Aβ in other regions appears to result from distinct mechanisms. This could involve unique neural processes linked to chronic pain as the anterior cingulate cortex and superior frontal cortex are primarily substrates involved in affective and cognitive processing of pain, respectively.^42,43^ Chronic pain may lead to functional and structural alterations in these regions,^43^ making them vulnerable to Aβ deposition independent of Alzheimer’s disease genetic risk and aging. Compared to our Aβ results, we did not find any differences in levels of tau tangle densities across chronic pain status.

Our findings contrast animal and human studies examining other chronic pain phenotypes with Alzheimer’s disease biomarkers. Using peripheral (sciatic) neuropathy to model chronic pain in mice, Guerreiro *et al*.^11^ found that induction of chronic neuropathic pain led to atrophy and increased levels of tau tangle density in the hippocampus compared to unaffected mice. Furthermore, knocking out tau-regulating genes removed the effect of chronic pain on hippocampal atrophy, confirming the neurodegenerative role of tau tangles in this pain model. Guerreiro *et al*.^11^ did not assess the impact of neuropathic pain on Aβ load, which may have also been elevated in mice with neuropathic pain. In a follow-up study, Wang *et al*.^44^ found that induction of neuropathic pain in mice was associated with upregulation of Aβ in the hippocampus and cerebral cortex in addition to greater phosphorylated tau.

Regarding human studies, Bell *et al.*^45^ found that chronic pain was associated with higher levels of plasma total tau and smaller hippocampal volume. Using data from the Alzheimer’s Disease Neuroimaging Initiative, Sadlon *et al*.^46^ found higher levels of total tau in people with chronic pain compared to no chronic pain. The present findings differ from other human studies by not finding an association of chronic pain with elevated tau tangle density. This inconsistency may be explained by major study differences. In contrast with prior human studies focusing on general chronic pain, we focused on MCP. Different chronic pain phenotypes may differentially influence dementia-related pathologies. Indeed, MCP is the one chronic pain phenotype causally linked to Alzheimer’s disease dementia specifically, for which Aβ is the key pathology.^12^ Furthermore, we focused on assessment of Alzheimer’s disease pathology directly in post-mortem brain tissue whereas Bell *et al*.^45^ and Sadlon *et al*.^46^ relied on extracerebral measures of Aβ and tau, which do not directly reflect levels of Alzheimer’s disease pathology in the brain. Extracerebral Aβ levels may be less precise in people with inflammatory conditions like chronic pain due to the facilitation of peripherally derived Aβ.^47,48^ Lastly, the present study assessed biomarkers of Alzheimer’s disease pathology at much later ages (*M* = 89.0, SD = 6.66) compared to Bell *et al*. (*M* = 67.06, SD = 2.61) and Sadlon *et al.* (*M* = 73.07, SD = 7.33). It is possible that chronic pain predominately influences tau levels in early older adulthood but accelerates Aβ accumulation as people age into their later 70 s and beyond. We also assessed the moderating role of *APOE*-ɛ4 allele, which was not assessed previously.

Interestingly, most of our significant findings were localized to MCP rather than SCP. This could be because MCP represents a pain state involving more severe underlying aetiology (systemic inflammation, widespread microglial activation, or greater dysregulation of the HPA axis), which leads to greater neural damage and amyloidosis.^49-57^ Such mechanisms would also explain why associations for MCP were mostly for people carrying an APOE-ɛ4 allele. APOE-ɛ4 increases pro-inflammatory cytokine production, reduces amyloid clearance and impairs blood–brain barrier integrity, all of which may amplify neural damage and Aβ accumulation related to the aetiology of MCP.^58-60^ Identifying biological mechanisms was beyond the scope of this study, but our findings suggest that understanding the aetiology of MCP, and how *APOE*-ɛ4 interacts therewith, may elucidate pathways to Alzheimer’s disease dementia.

Our findings should be taken into the context of limitations and strengths. First, we cannot directly address causality due to the cross-sectional observational nature of our study. Reverse causation is possible as people with cognitive decline are at greater risk for pain-inducing injuries.^61^ However, studies using Mendelian randomization and animal modelling provide evidence that chronic pain leads to cognitive decline and lower hippocampal volume, typical indicators of Alzheimer’s disease risk.^11,12^ Second, our MCP phenotype differs from previous work^15^ as our measure only assessed chronic musculoskeletal pain. Zhao *et al*.^15^ included visceral pain, face pain and headaches, which may lead to a different pattern of results. However, MCP mostly involved musculoskeletal pain in their study (>70%).

Regarding strengths, our study involved a large sample of older adults that had considerable racial and ethnic representation (MAP). Our analyses of cognitive decline also spanned up to 30 years, which is the longest any study has assessed chronic pain and cognitive decline to our knowledge. Lastly, our measurement of Alzheimer’s disease pathology was done using direct immunoassaying of post-mortem tissue, the most direct level of evidence. Prior studies so far have relied on plasma and CSF biomarkers.^45,46^

In conclusion, our study highlights the need to consider MCP as a significant risk factor for cognitive decline and Alzheimer’s disease. Understanding the mechanisms behind this association, particularly in the context of *APOE*-ɛ4, is crucial for developing targeted interventions to mitigate the impact of chronic pain on cognitive function and Alzheimer’s disease progression. Further research, especially longitudinal studies, is needed to explore the temporal relationship between MCP and Alzheimer’s disease-related changes, as well as the potential influences of genetic and environmental factors. Addressing MCP in the context of Alzheimer’s disease could lead to new therapeutic strategies that preserve cognitive function and improve the quality of life for affected individuals.

## Supplementary Material

fcaf208_Supplementary_Data

## Data Availability

Data are available for research access (www.radc.rush.edu). Statistical code and full output is freely accessible (https://github.com/trbellucsd/ROSMAP_Pain_AD_Risk).
