# Supplementary material for: Multisite chronic pain: association with cognitive decline and post-mortem Alzheimer’s biomarkers
Source: Brain Commun. 2025 May 28;7(3):fcaf208. doi: 10.1093/braincomms/fcaf208 (PMC12149845; doi:10.1093/braincomms/fcaf208)
Supplement: fcaf208_Supplementary_Data [file fcaf208_supplementary_data.docx]

| **List of Supplemental Material** |
| --- |
|  |
| eTable 1. Mixed model examining associations of chronic pain, time, and *APOE*-ε4 load on processing speed, semantic memory, and perceptual orientation (n=3459). |
| eTable 2. Association of chronic pain with likelihood of Alzheimer’s type dementia at final assessment (n=3459). |
| eTable 3. Association of multisite chronic pain versus no chronic pain with β-Amyloid load and tangles part 1. |
| eTable 4. Association of multisite chronic pain versus no chronic pain with β-Amyloid load and tangles part 2. |
| eFigure 1. Levels of tangles in the entorhinal cortex and the hippocampus based on chronic pain and *APOE*-ε4 allele status. |

| eTable 1. Mixed model examining associations of chronic pain, time, and *APOE*-ε4 load on processing speed, semantic memory, and perceptual orientation (*n*=3459). | | | | | | | | | |
| --- | --- | --- | --- | --- | --- | --- | --- | --- | --- |
|  | Processing Speed | | | Semantic Memory | | | Perceptual Orientation | | |
| Model Term | b | SE | p | b | SE | p | b | SE | p |
| Time (Time-Varying Age) | **-.06** | **.001** | **<.001** | **-.03** | **.001** | **<.001** | **-.02** | **.001** | **<.001** |
| Main Effects (Baseline Differences) |  |  |  |  |  |  |  |  |  |
| MCP (vs. No CP) | .02 | .049 | .722 | .03 | .04 | .467 | -.07 | .05 | .101 |
| SCP (vs. No CP) | -.05 | .042 | .210 | **.10** | **.04** | **.008** | .01 | .04 | .734 |
| MCP (vs. SCP) | .07 | .059 | .237 | -.07 | .05 | .204 | -.09 | .05 | .11 |
| *APOE*-ε4 load | **-.15** | **.028** | **<.001** | **-.10** | **.02** | **<.001** | **-.08** | **.03** | **.002** |
| Interactions with *APOE*-ε4 Load |  |  |  |  |  |  |  |  |  |
| MCP (vs. No CP) | .12 | .085 | .17 | **.16** | **.07** | **.030** | .21 | .08 | .008 |
| SCP (vs. No CP) | .12 | .070 | .081 | .01 | .06 | .817 | -.01 | .06 | .856 |
| MCP (vs. SCP) | -.01 | .103 | .959 | .15 | .09 | .102 | **.22** | **.09** | **.021** |
| Interactions with Time |  |  |  |  |  |  |  |  |  |
| MCP (vs. No CP) | .01 | .002 | .916 | .00 | .002 | .569 | .00 | .003 | .077 |
| SCP (vs. No CP) | .003 | .002 | .228 | .00 | .002 | .943 | .00 | .002 | .096 |
| MCP (vs. SCP) | -.003 | .003 | .338 | .00 | .003 | .614 | .00 | .003 | .845 |
| *APOE*-ε4 load*Time | **-.01** | **.001** | **<.001** | **-.02** | **.001** | **<.001** | **-.01** | **.002** | **<.001** |
| Interactions with *APOE*-ε4 Load and Time |  |  |  |  |  |  |  |  |  |
| MCP (vs. No CP) | **-.01** | **.005** | **.001** | .00 | .004 | .447 | **-.01** | **.005** | **.004** |
| SCP (vs. No CP) | -.002 | .003 | .489 | **.01** | **.003** | **<.001** | .00 | .004 | .296 |
| MCP (vs. SCP) | **-.01** | **.005** | **.021** | **-.01** | **.005** | **.003** | **-.02** | **.006** | **.002** |
| Note. *APOE*-ε4=apolipoprotein epsilon 4 allele; CP = chronic pain; MCP = multisite chronic pain; SCP = single-site chronic pain. Models were adjusted for the effect of age at baseline, sex, education, race, analgesic use, number of total follow-ups completed, depressive symptoms, history of stroke, history of congestive heart failure, and history of diabetes. Bolded values indicate statistically significant associations. | | | | | | | | | |

| eTable 2. Association of chronic pain with likelihood of Alzheimer’s type dementia at final assessment (n=3459). | | | |
| --- | --- | --- | --- |
|  | AD Dementia | | |
| Model Term | OR | SE | p |
| Main Effects |  |  |  |
| MCP (vs. No CP) | **1.65** | **.179** | **.005** |
| SCP (vs. No CP) | 1.16 | .144 | .312 |
| MCP (vs. SCP) | 1.43 | .213 | .095 |
| *APOE-*ε4 load | **.51** | **.086** | **<.001** |
| Interaction with *APOE-*ε4 load |  |  |  |
| MCP (vs. No CP) | 1.12 | .278 | .682 |
| SCP (vs. No CP) | .88 | .216 | .565 |
| MCP (vs. SCP) | 1.27 | .330 | .471 |
| Note. AD = Alzheimer’s disease; *APOE*-ε4=apolipoprotein epsilon 4 allele; CP = chronic pain; MCP = multisite chronic pain; SCP = single-site chronic pain. Models were adjusted for the effect of age at final assessment, age at baseline, sex, education, race, analgesic use, number of total follow-ups completed, depressive symptoms, history of stroke, history of congestive heart failure, and history of diabetes. Bolded values indicate statistically significant associations. | | | |

| eTable 3. Association of multisite chronic pain versus no chronic pain with β-Amyloid load and tangles part 1. | | | | | | | | | | | | |
| --- | --- | --- | --- | --- | --- | --- | --- | --- | --- | --- | --- | --- |
| Model Term | Aβ in IT (n=910) | | | Aβ in AG (n=919) | | | Aβ in MF (n=922) | | | Aβ in Calc (n=918) | | |
| Main Effects | b | SE | p | b | SE | p | b | SE | p | b | SE | p |
| MCP (vs. No CP) | **.84** | **.38** | **.028** | .76 | .48 | .112 | .89 | .50 | .078 | .37 | .28 | .191 |
| SCP (vs. No CP) | -.34 | .33 | .308 | -.40 | .42 | .343 | -.60 | .44 | .178 | -.22 | .25 | .388 |
| MCP (vs. SCP) | **1.18** | **.46** | **.011** | **1.16** | **.59** | **.047** | **1.49** | **.61** | **.016** | .59 | .35 | .091 |
| *APOE*-ε4 load | **1.85** | **.22** | **<.001** | **2.12** | **.28** | **<.001** | **2.21** | **.29** | **<.001** | **1.59** | **.16** | **<.001** |
| Interaction with Age at Death |  |  |  |  |  |  |  |  |  |  |  |  |
| *APOE*-ε4 load | -.16 | .21 | .459 | -.08 | .26 | .770 | -.03 | .27 | .907 | -.04 | .15 | .802 |
| MCP (vs. No CP) | .64 | .37 | .086 | .70 | .47 | .138 | .73 | .50 | .142 | **.64** | **.28** | **.022** |
| SCP (vs. No CP) | -.49 | .32 | .128 | -.44 | .41 | .285 | -.69 | .43 | .108 | -.16 | .24 | .521 |
| MCP (vs. SCP) | **1.14** | **.46** | **.013** | 1.14 | .58 | .050 | **1.42** | **.61** | **.020** | **.79** | **.34** | **.022** |
| Interaction with *APOE*-ε4 load |  |  |  |  |  |  |  |  |  |  |  |  |
| MCP (vs. No CP) | 1.01 | .69 | .147 | 1.54 | .87 | .077 | 1.03 | .92 | .262 | .63 | .51 | .224 |
| SCP (vs. No CP) | .59 | .53 | .273 | .04 | .67 | .956 | -.30 | .71 | .676 | .37 | .40 | .354 |
| MCP (vs. SCP) | .42 | .82 | .605 | 1.51 | 1.03 | .143 | 1.33 | 1.08 | .221 | .26 | .61 | .669 |
| Interaction with *APOE*-ε4 load and Age at Death | | |  |  |  |  |  |  |  |  |  |  |
| MCP (vs. No CP) | **1.48** | **.74** | **.045** | .65 | .93 | .486 | 1.09 | .98 | .266 | **1.13** | **.55** | **.039** |
| SCP (vs. No CP) | -.23 | .59 | .693 | -.04 | .73 | .954 | -.08 | .77 | .922 | -.31 | .44 | .476 |
| MCP (vs. SCP) | 1.71 | .90 | .057 | .69 | 1.13 | .542 | 1.17 | 1.19 | .372 | **1.44** | **.67** | **.031** |
|  | Tangles in IT (n=1004) | | | Tangles in AG (n=1009) | | | Tangles in MF (n=1013) | | | Tangles in Calc (n=999) | | |
| Main Effects | b | SE | p | b | SE | p | b | SE | p | b | SE | p |
| MCP (vs. No CP) | -.05 | 1.33 | .970 | -.59 | .63 | .349 | -.53 | .58 | .364 | -.22 | .22 | .314 |
| SCP (vs. No CP) | -.51 | 1.16 | .657 | -.79 | .55 | .154 | -.61 | .51 | .232 | -.18 | .19 | .354 |
| MCP (vs. SCP) | .46 | 1.62 | .774 | .20 | .77 | .797 | .08 | .71 | .907 | -.04 | .27 | .875 |
| *APOE*-ε4 load | **4.62** | **.76** | **<.001** | **1.83** | **.36** | **<.001** | **1.58** | **.33** | **<.001** | **.44** | **.13** | **<.001** |
| Interaction with Age at Death |  |  |  |  |  |  |  |  |  |  |  |  |
| *APOE*-ε4 load | .38 | .72 | .603 | -.62 | .33 | .059 | -.31 | .31 | .316 | -.06 | .12 | .577 |
| MCP (vs. No CP) | 1.45 | 1.28 | .258 | .44 | .61 | .466 | .64 | .57 | .259 | -.06 | .21 | .772 |
| SCP (vs. No CP) | -.22 | 1.11 | .843 | -.04 | .53 | .933 | .11 | .50 | .827 | -.07 | .19 | .708 |
| MCP (vs. SCP) | 1.67 | 1.57 | .287 | .49 | .75 | .513 | .53 | .70 | .448 | .01 | .26 | .973 |
| Interaction with *APOE*-ε4 load |  |  |  |  |  |  |  |  |  |  |  |  |
| MCP (vs. No CP) | .34 | 2.32 | .883 | -.07 | 1.09 | .947 | .75 | 1.01 | .457 | .25 | .39 | .525 |
| SCP (vs. No CP) | .04 | 1.86 | .985 | .82 | .88 | .349 | **2.28** | **.82** | **.005** | -.39 | .31 | .214 |
| MCP (vs. SCP) | .31 | 2.78 | .912 | -.90 | 1.31 | .494 | -1.53 | 1.21 | .207 | .63 | .46 | .174 |
| Interaction with *APOE*-ε4 load and Age at Death | | |  |  |  |  |  |  |  |  |  |  |
| MCP (vs. No CP) | 1.80 | 2.61 | .491 | .96 | 1.23 | .436 | .43 | 1.14 | .706 | -.11 | .43 | .797 |
| SCP (vs. No CP) | -2.73 | 2.00 | .174 | -1.47 | .94 | .118 | **-3.25** | **.87** | **<.001** | .20 | .33 | .557 |
| MCP (vs. SCP) | 4.53 | 3.13 | .149 | 2.43 | 1.48 | .101 | **3.68** | **1.37** | **.007** | -.31 | .52 | .556 |
| Note. AG = angular gyrus; *APOE*-ε4=apolipoprotein epsilon 4 allele; Calc = calcarine cortex; CP = chronic pain; IT = inferior temporal; MCP = multisite chronic pain; MF = midfrontal cortex; SCP = single-site chronic pain. Models were adjusted for the effect of age at death, age at baseline, interval between death and autopsy, interval between last assessment and death, final diagnosis, sex, education, race, analgesic use, number of total follow-ups completed, depressive symptoms, history of stroke, history of congestive heart failure, and history of diabetes. Bolded values indicate statistically significant associations. | | | | | | | | | | | | |

| eTable 4. Association of multisite chronic pain versus no chronic pain with β-Amyloid load and tangles part 2. | | | | | | | |
| --- | --- | --- | --- | --- | --- | --- | --- |
|  | Aβ in CG (n=865) | | | Aβ in SF (n=865) | | |  |
| Main Effects | b | SE | p | b | SE | p |  |
| MCP (vs. No CP) | **1.02** | **.49** | **.039** | **1.05** | **.44** | **.017** |  |
| SCP (vs. No CP) | -.67 | .43 | .120 | -.53 | .39 | .174 |  |
| MCP (vs. SCP) | **1.70** | **.60** | **.005** | **1.58** | **.54** | **.003** |  |
| *APOE*-ε4 load | **1.91** | **.29** | **<.001** | **1.95** | **.26** | **<.001** |  |
| Interaction with Age at Death |  |  |  |  |  |  |  |
| *APOE*-ε4 load | -.06 | .27 | .822 | -.07 | .24 | .786 |  |
| MCP (vs. No CP) | .79 | .49 | .106 | .82 | .43 | .058 |  |
| SCP (vs. No CP) | -.33 | .42 | .427 | -.51 | .38 | .180 |  |
| MCP (vs. SCP) | 1.12 | .59 | .059 | **1.33** | **.53** | **.013** |  |
| Interaction with *APOE*-ε4 load |  |  |  |  |  |  |  |
| MCP (vs. No CP) | .78 | .89 | .377 | .51 | .79 | .517 |  |
| SCP (vs. No CP) | .09 | .69 | .899 | .13 | .62 | .833 |  |
| MCP (vs. SCP) | .70 | 1.05 | .508 | .38 | .93 | .685 |  |
| Interaction with *APOE*-ε4 load and Age at Death | | |  |  |  |  |  |
| MCP (vs. No CP) | 1.02 | .94 | .281 | 1.09 | .84 | .193 |  |
| SCP (vs. No CP) | -.44 | .75 | .559 | -.17 | .67 | .801 |  |
| MCP (vs. SCP) | 1.45 | 1.14 | .205 | 1.26 | 1.02 | .217 |  |
|  | Tangles in CG (n=982) | | | Tangles in SF (n=958) | | |  |
| Main Effects | b | SE | p | b | SE | p |  |
| MCP (vs. No CP) | -.91 | .67 | .175 | -.43 | .69 | .537 |  |
| SCP (vs. No CP) | -.81 | .59 | .169 | -.47 | .61 | .435 |  |
| MCP (vs. SCP) | -.10 | .82 | .903 | .05 | .84 | .956 |  |
| *APOE*-ε4 load | **1.90** | **.38** | **<.001** | **1.88** | **.40** | **<.001** |  |
| Interaction with Age at Death |  |  |  |  |  |  |  |
| *APOE*-ε4 load | .30 | .36 | .411 | -.20 | .37 | .591 |  |
| MCP (vs. No CP) | .74 | .65 | .256 | .40 | .67 | .548 |  |
| SCP (vs. No CP) | -.18 | .57 | .751 | -.33 | .58 | .569 |  |
| MCP (vs. SCP) | .92 | .80 | .250 | .73 | .81 | .369 |  |
| Interaction with *APOE*-ε4 load |  |  |  |  |  |  |  |
| MCP (vs. No CP) | -.53 | 1.16 | .645 | -.18 | 1.19 | .880 |  |
| SCP (vs. No CP) | -.05 | .96 | .962 | .43 | .98 | .661 |  |
| MCP (vs. SCP) | -.49 | 1.41 | .729 | -.61 | 1.44 | .672 |  |
| Interaction with *APOE*-ε4 load and Age at Death | | |  |  |  |  |  |
| MCP (vs. No CP) | .72 | 1.31 | .583 | .52 | 1.34 | .697 |  |
| SCP (vs. No CP) | **-2.28** | **1.01** | **.024** | -1.03 | 1.03 | .319 |  |
| MCP (vs. SCP) | 3.00 | 1.58 | .057 | 1.55 | 1.61 | .336 |  |
| Note. *APOE*-ε4=apolipoprotein epsilon 4 allele; CG = anterior cingulate cortex; CP = chronic pain; MCP = multisite chronic pain; SCP = single-site chronic pain; SF = superior frontal cortex. Models were adjusted for the effect of age at death, age at baseline, interval between death and autopsy, interval between last assessment and death, final diagnosis, sex, education, race, analgesic use, number of total follow-ups completed, depressive symptoms, history of stroke, history of congestive heart failure, and history of diabetes. Bolded values indicate statistically significant associations. | | | | | | | |


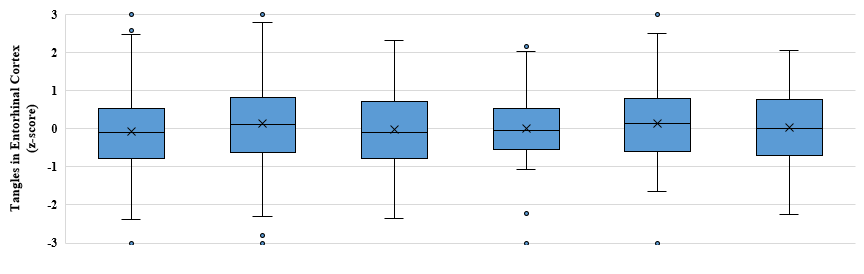

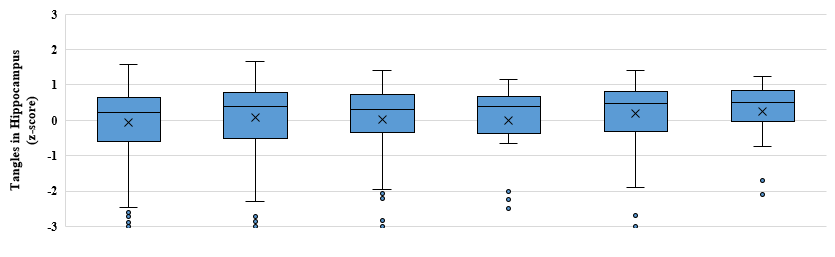


**B.**

**A.**

**MCP/ε4+**

**MCP/ε4-**

**SCP/ε4+**

**SCP/ε4-**

**CP/ε4-**

**CP/ε4+**

**CP/ε4-**

**CP/ε4+**

**SCP/ε4-**

**SCP/ε4+**

**MCP/ε4-**

**MCP/ε4+**

eFigure 1. Levels of tangles in the entorhinal cortex (A) and the hippocampus (B) based on chronic pain and *APOE*-ε4 allele status. Note. CP = chronic pain; ε4+ = presence of ε4 allele; ε4- = absence of ε4 allele; MCP = multisite chronic pain; SCP = single-site chronic pain. Linear mixed models revealed no significant differences by chronic pain status in tau tangle densities in the entorhinal cortex and hippocampus (p’s<.05).
